# Herbal Formulation C168 Attenuates Proliferation and Induces Apoptosis in HCT 116 Human Colorectal Carcinoma Cells: Role of Oxidative Stress and DNA Damage

**DOI:** 10.1155/2016/2091085

**Published:** 2016-01-17

**Authors:** Lek Mun Leong, Kok Meng Chan, Asmah Hamid, Jalifah Latip, Nor Fadilah Rajab

**Affiliations:** ^1^Biomedical Science Programme, School of Diagnostic and Applied Health Science, Faculty of Health Sciences, Universiti Kebangsaan Malaysia, Jalan Raja Muda Abdul Aziz, 50300 Kuala Lumpur, Malaysia; ^2^Environmental Health and Industrial Safety Programme, School of Diagnostic and Applied Health Science, Faculty of Health Sciences, Universiti Kebangsaan Malaysia, Jalan Raja Muda Abdul Aziz, 50300 Kuala Lumpur, Malaysia; ^3^Toxicology Laboratory, Faculty of Health Sciences, Universiti Kebangsaan Malaysia, Jalan Raja Muda Abdul Aziz, 50300 Kuala Lumpur, Malaysia; ^4^School of Chemical Science and Food Technology, Faculty of Science and Technology, Universiti Kebangsaan Malaysia, Jalan Reko, 43600 Bangi, Selangor, Malaysia

## Abstract

The use of herbal formulations has gained scientific interest, particularly in cancer treatment. In this study, the herbal formulation of interest, denoted as C168, is a mixture of eight genera of plants. This study aims to investigate the antiproliferative effect of C168 methanol extract (CME) on various cancer cells and its underlying mechanism of action on the most responsive cell line, namely, HCT 116 cells. CME exerted antiproliferative activities on HCT 116 colorectal carcinoma cells and HepG2 hepatocellular carcinoma cells but not on CCD-841-CoN normal colon epithelial cells, Jurkat E6.1 lymphoblastic leukemic cells, and V79-4 Chinese hamster lung fibroblasts. Further investigation on HCT 116 cells showed that CME induced G_2_/M cell-cycle arrest and apoptosis. Treatment of CME induced oxidative stress in HCT 116 cells by increasing the superoxide anion level and decreasing the intracellular glutathione. CME also increased tail moment value and H2AX phosphorylation in HCT 116 cells, suggesting DNA damage as an early signal of CME induced apoptosis. Loss of mitochondrial membrane potential in CME-treated cells also indicated the involvement of mitochondria in CME induced apoptosis. This study indicated the selectivity of CME toward colon cancer cells with the involvement of oxidative damage as its possible mechanism of action.

## 1. Introduction

Traditional Chinese medicine (TCM) is an important component in complementary and alternative medicine. TCM has evolved for a thousand years on the basis of its unique system of theories. One of the principal theories proposed in the classic TCM text* Yellow Emperor's Internal Cannon* was the balance between vital* qi* and pathogenic* qi* in the human body [1]. This principle has been widely used in designing formulations to treat various diseases. TCM has gained increasing scientific interest in cancer treatment over the years. The role of TCM in the following three aspects of cancer therapy has been extensively explored: prevention of tumorigenesis, reduction of side effects and enhancement of efficacy of conventional therapy, and reduction of tumor recurrence and metastasis [1].

Among various forms of TCM therapies, such as herbal medicine, acupoint stimulation, massage, TCM psychological intervention, and* qigong*, herbal medicine is the most frequently applied TCM therapy for cancer patients [2]. Herbal formulation is a mixture of herbs designed for a specific condition or disease. Examples of herbal formulations with scientific evidence in the treatment of colon cancer include* Pi-Sheng* decoction,* Yi-Qi-Zhu-Yu* decoction,* Jian-Pi-Xiao-Liu* decoction,* Jian-Qi-Jie-Du* decoction,* Jian-Pi-Yi-Qi* decoction, and* Fu-Pi-Yi-Wei* decoction [3]. Combination of multiple drugs as a formulation provides the advantage of targeting multiple mechanisms in a disease to enhance treatment [4].

Colorectal cancer is a common malignant neoplasm prevalent in both developed and developing countries [5, 6]. This disease ranks second among causes of cancer-related deaths worldwide, comprising 10%–15% of all forms of cancer [7]. Cancer led to 7.6 million deaths globally in 2008 and remains a healthcare burden in terms of managing the disease [8].

Chemotherapy agents used for colorectal cancer include 5-fluorouracil (5-FU), capecitabine, leucovorin, and oxaliplatin [7]. Each chemotherapeutic agent demonstrates a distinct mechanism of action. For instance, 5-FU, a commonly used chemotherapeutic agent, and its prodrug capecitabine exert antiproliferative effects by generating thymidylate stress [7]. Leucovorin is often incorporated into the 5-FU regimen as a combinatorial treatment to enhance the clinical effects of 5-FU by serving as a substrate to form N^5^,N^10^-methylene tetrahydrofolate (CH_2_H_4_PteGlu). N^5^,N^10^-Methylene tetrahydrofolate serves as a rate-limiting cofactor in 5-FU inhibition of thymidylate synthesis. Oxaliplatin is a platinum-based cytotoxic agent that forms DNA-platinum adducts to inhibit cell growth [9]. Current clinical approaches to colorectal cancer focus on combinatorial regimens, such as the Mayo Clinic regimen, de Gramont regimen, modified de Gramont regimen, and FOLFOX [10]. Therefore, C168 was formulated through the combination of different herbs to achieve the beneficial effect of a combinatorial regimen.

However, most cancer treatments remain inadequate and far from desired perfection [11]. Commonly used chemotherapeutic agents are often associated with multiple side effects of the treatment dose [12]. Chemotherapy drugs such as oxaliplatin, etoposide, and 5-FU cause peripheral neuropathy, myelosuppression, and leukopenia, respectively [13–15]. To address these issues, studies are conducted on the combination of multiple natural products or of natural products with conventional drugs to enhance the therapeutic effect of these drugs, with the hope of reducing their side effects [16–19]. One of the combinations that underwent research was the combination of notoginseng extract with 5-FU to enhance the efficacy of 5-FU in colorectal cancer cell lines [16].

In the present study, the herbal formulation of interest, denoted as C168, contained eight different genera of plant which include* Cinnamomum* spp.,* Zingiber* spp.,* Atractylodes* spp.,* Carthamus* spp.,* Angelica* spp.,* Curcuma* spp.,* Glycyrrhiza* spp., and* Astragalus* spp. The formulation and species of herbs were not disclosed for the reasons related to intellectual property protection. Several genera found in herbal formulation C168 have been recognized to exert antiproliferative effects on colorectal cancer cell lines individually. These genera include* Cinnamomum* spp.,* Zingiber* spp., and* Astragalus* spp. [20–25]. An anecdotal report claimed that the herbal formulation C168 relieved the symptoms of colon cancer and served as a potential natural product for colon cancer treatment. However, no scientific evidence has been presented to validate these claims. Therefore, the current study focuses on the antiproliferative effect of C168 methanol extract (CME), as well as its underlying mechanism of action.

## 2. Materials and Methods

### 2.1. Chemicals and Cell Lines

All chemicals were purchased from Sigma (USA) unless stated otherwise. HCT 116 human colorectal carcinoma cells, CCD-841-CoN normal colon epithelial cells, Jurkat E6.1 lymphoblastic leukemic cells, HepG2 hepatocellular carcinoma cells, and V79-4 Chinese hamster lung fibroblasts were obtained from American Type Culture Collection (Rockville, MD USA). CCD-841-CoN and V79-4 cells were maintained in Dulbecco's Modified Eagle's Medium (DMEM), whereas HepG2 and Jurkat E6.1 cells were maintained in Eagle Minimum Essential Medium (EMEM) and RPMI 1640 medium, respectively. All mediums were purchased from Gibco Invitrogen, USA, and supplemented with 10% fetal bovine serum (PAA Laboratories, GmbH) and 100 U/mL of Penicillin-Streptomycin (PAA Laboratories, GmbH). Cells were cultured at 37°C in 5% CO_2_ condition at the Toxicology Laboratory, Faculty of Health Science, Universiti Kebangsaan Malaysia.

### 2.2. Preparation of C168 Methanol Extract (CME)

C168 in powder form was provided by Inchoice Technology Sdn. Bhd. (Selangor, Malaysia). C168 consisted of seven genera of Chinese herbs, namely,* Cinnamomum* spp.,* Zingiber* spp.,* Atractylodes* spp.,* Carthamus* spp.,* Angelica* spp.,* Curcuma* spp.,* Glycyrrhiza* spp., and* Astragalus* spp. C168 powder was macerated with methanol (Merck, Malaysia) for 72 hours and filtered using a Whatman filter paper. The solvent was dried by rotary evaporation, followed by freeze drying. The extract was then reconstituted in DMSO (Ajax Finechem, Thermo Fisher Scientific) to prepare 800 mg/mL stock solution for* in vitro* testing.

### 2.3. Cell Plating and Treatment* In Vitro*

HCT 116, V79-4, HepG2, and CCD-841-CoN cells were plated at a density of 2 × 10^4^/cm^2^, whereas Jurkat E6.1 cells were plated at a seeding concentration of 1 × 10^6^ cells/mL. Cells were allowed to stand overnight for cell attachment. CME treatment was administered in a concentration-dependent or time-dependent manner as indicated in each subsection.

### 2.4. Cell Viability Assay

MTT [3-(4,5-dimethyl-2-thiazolyl)-2,5-diphenyl-2H-tetrazolium bromide] assay was performed to determine the cytotoxicity effect of CME on a panel of cell lines, namely, HCT 116, CCD-841-CoN, HepG2, Jurkat E6.1, and V79-4 cells, as previously described [26]. Plating was performed in a 96-well plate and treated with CME for 24 hours. MTT solution (5 mg/mL) was added following incubation at a final concentration of 0.5 mg/mL and further incubated for 4 hours. Supernatant was removed, and DMSO was added to dissolve the formed formazan crystal. The plate was further incubated for 15 minutes prior to measurement at 570 nm with an ELISA plate reader (Bio-Rad, USA). Cell viability was calculated as follows:

(1) $$\begin{array}{c} \%\;\;viability=\frac{\text{optical density of sample}}{\text{optical density of control}}\times 100\%. \end{array}$$

The cell line with the most potent IC_50_ was selected for further evaluation for the possible CME mechanism of action.

### 2.5. Annexin V-FITC/PI Labeling Assay

The mode of cell death induced by CME was assessed as previously described [27]. On the basis of the IC_50_ obtained by MTT assay, HCT 116 cells were selected as the model to be used for the present and subsequent studies. HCT 116 cells were treated with various concentrations of CME for 72 hours. The CME-treated HCT 116 cells were harvested and then washed with chilled PBS prior to suspension in Annexin binding buffer (ABB) at 1 × 10^6^ cells/mL. Subsequently, 100 *μ*L of cell suspension was transferred to a polystyrene round-bottom tube and stained with 5 *µ*L of Annexin V-FITC (BD Bioscience, USA) for 15 minutes and then with 5 *µ*L of propidium iodide (50 *µ*g/mL) for 2 minutes. Staining was performed in dark at room temperature. Afterward, 400 *µ*L of ABB was added to the stained cells prior to analysis with FACSCanto II flow cytometer (BD Bioscience, USA).

### 2.6. Trypan Blue Dye Exclusion Test

The total number of cells was assessed using the trypan blue dye exclusion test. HCT 116 cells were treated with various concentrations of CME for 24 hours before they were harvested. Cells were centrifuged (2500 rpm, 5 minutes, 4°C) before 3 mL of complete culture media was added. Subsequently, 100 *μ*L of cell suspension was added to 400 *µ*L of 0.4% trypan blue reagent and then mixed thoroughly. Cell count was assessed using a hemocytometer. Under a light microscope, the cells that picked up trypan blue dye were identified as dead cells, whereas those that excluded trypan blue were identified as viable cells. The number of viable cells and that of dead cells were calculated and recorded.

### 2.7. Cell-Cycle Analysis

Cell-cycle distribution was determined as previously described [28]. Briefly, 5 × 10^5^ CME-treated cells were washed with 30 mL of chilled PBS, centrifuged (2500 rpm, 5 minutes, 4°C), fixed with 70% ethanol overnight, and then centrifuged again. Subsequently, 1 × 10^5^ cells were stained with 500 *µ*L of PI solution (BD Bioscience, USA) for 15 minutes at room temperature. Stained cells were analyzed using FACSCanto II flow cytometer (BD Bioscience, USA) installed with ModFit LT (Verity Software House).

### 2.8. Assessment of Reactive Oxygen Species

Reactive oxygen species (ROS) content was assessed by hydroethidine staining as previously described [27]. C168 treatment was administered at different time-point intervals before HCT 116 cells were harvested. CME-treated cells were washed with chilled PBS and then suspended in serum-free medium containing 1 *µ*L of 10 mM hydroethidine (Gibco, Invitrogen). Staining was performed in dark (30 minutes, 37°C), and the cells were washed prior to suspension in 500 *µ*L of chilled PBS and analysis with FACSCanto II flow cytometry (BD Bioscience, USA).

### 2.9. Determination of Intracellular Free Thiol Level

Intracellular free thiol level was determined as previously described [29]. CME-treated cells were washed with chilled PBS, and 100 *µ*L of chilled lysis buffer (50 mM K_2_HPO_4_, 1 mM EDTA, pH 6.5, and 0.1% v/v Triton-X-100) was added. Cells were incubated (4°C, 15 minutes) for lysis process to occur, followed by centrifugation (12 000 rpm, 15 minutes, 4°C). Supernatant was collected as protein lysate. Protein concentration was determined using Bradford protein assay, whereas the glutathione level was assessed using Ellman assay. The standard curve of glutathione was generated using a set of standard solutions ranging from 1.25 mM to 4.88 *µ*M. This step was performed by reconstituting the reduced glutathione with a reaction buffer at pH 6.5 (0.1 M Na_2_HPO_4_·7H_2_O and 1 mM EDTA, pH 6.5). Subsequently, 50 *µ*L of the sample was added into a 96-well plate, followed by 40 *µ*L of reaction buffer at pH 8 (0.1 M Na_2_HPO_4_·7H_2_O and 1 mM EDTA, pH 8) and 10 *µ*L of dithiobis(2-nitrobenzoic acid) (DTNB) (4 mg/mL). Reaction mixtures were incubated for 15 minutes, and absorbance was read at 415 nm. The concentration of free thiol in cell lysate was calculated and expressed as nmol glutathione/mg protein.

### 2.10. Alkaline Comet Assay

Alkaline comet assay was performed as previously described to access DNA damage induced by CME [30]. CME-treated HCT 116 cells were harvested and washed twice with Ca^2+^, Mg^2+^-free PBS. Cell pellets were then mixed thoroughly with 0.6% low melting point agarose and laid on hardened 0.6% normal melting agarose. The agarose was allowed to solidify and subsequently placed in a chilled lysis buffer (2.5 M NaCl, 100 mM EDTA, 10 mM Tris, and 1% Triton-X) for lysis to occur. Slides were then incubated in an electrophoresis buffer (0.3 N NaOH, 1 mM EDTA) for 20 minutes to facilitate DNA unwinding. Electrophoresis was performed under 25 V, 300 mA for 20 minutes. Subsequently, slides were rinsed with neutralizing buffer (400 mM Tris) thrice prior to staining with 10 *µ*g/mL ethidium bromide solution. Slides were kept overnight before they were observed under fluorescence microscope (Olympus, Japan) and then analyzed with CometScore (Tritek Corp., USA).

### 2.11. Assessment of Mitochondrial Membrane Potential

Mitochondrial membrane potential (MMP) was assessed as previously described [31]. CME-treated HCT 116 cells were suspended in 1 mL of serum-free media, added with 1 *µ*L of 50 *µ*M tetramethylrhodamine ethyl ester (TMRE) (Molecular Probes, Invitrogen). The cells were incubated in the dark for staining purposes (15 minutes, 37°C), followed by centrifugation (2500 rpm, 5 minutes, 4°C). The stained cells were washed with chilled PBS once prior to suspension in 500 *µ*L of PBS and then analyzed with FACSCanto II flow cytometer (BD Bioscience, USA).

### 2.12. Western Immunoblot Analysis

Western blot analysis was performed as previously described to access the level of phosphorylated H2AX [32]. HCT 116 cells were treated with 500 *µ*g/mL CME at various time points and then harvested via scrapping. Cell lysates were collected, and protein samples were denatured at 95°C for 5 minutes. Subsequently, 20 *µ*g/mL protein lysate was resolved in 15% SDS-polyacrylamide gel and blotted on a polyvinylidene fluoride membrane. Rabbit phosphorylated H2AX antibody was added to the blot and then allowed to incubate at room temperature for 2 hours. Meanwhile, rabbit monoclonal beta-actin was used to detect beta-actin as loading control. Following incubation of primary antibody, a secondary antibody (goat anti-rabbit HRP conjugated antibody) was added and incubated at room temperature for 1 hour prior to detection by chemiluminescence method. All antibodies were purchased from Cell Signaling Technology (Massachusetts, USA).

### 2.13. Phytochemical Screening

Phytochemical screening was performed using the techniques described below [33, 34].

#### 2.13.1. Alkaloid Screening

Up to 500 mg of CME was added into chloroform until a thick slurry was formed. Thereafter, 10 mL of ammonium chloroform was added into the mixture and filtered into a test tube. Following this step, 1 mL of 2 M sulfuric acid was added and allowed to stand for 5 minutes. An aqueous layer was obtained, and Mayer's reagent was added. Any precipitation indicated the presence of alkaloid.

#### 2.13.2. Triterpenoid Screening

Up to 500 mg of CME was added into 4 mL of acetic anhydride and heated to boiling temperature. The extract was then cooled, and 1 mL of concentrated sulfuric acid was added along the side of the test tube. Formation of pink color indicated the presence of triterpenoid.

#### 2.13.3. Phenolic Compound Screening

CME was dissolved in 70% ethanol. Equal volume of 5% iron(III) chloride solution was added into the extract and then mixed. A deep bluish green solution indicated the presence of phenolic compounds.

#### 2.13.4. Saponin Screening

Up to 2 mL of distilled water was added to 2 mL of the extract and then shaken vigorously. Stable persistent froth indicated the presence of saponin.

### 2.14. Statistical Analysis

Statistical Package of the Social Science (SPSS) version 20 (IBM Corp., New York, USA) was used for statistical analysis. Data were expressed as means ± standard error of mean (SEM). The results of Annexin V-FITC/PI labeling assay, trypan blue dye exclusion test, cell-cycle analysis, ROS content analysis, intracellular free thiol level determination, alkaline comet assay, and MMP assessment were all analyzed using one-way ANOVA. Dunnett's* post hoc* test was used to identify significant treatment effects (*p* < 0.05) for the abovementioned assay. A value of *p* < 0.05 was considered statistically significant.

## 3. Results

### 3.1. CME Exerted the Highest Antiproliferative Effect on HCT 116 Colorectal Carcinoma Cells* In Vitro*

**Figure 1:**
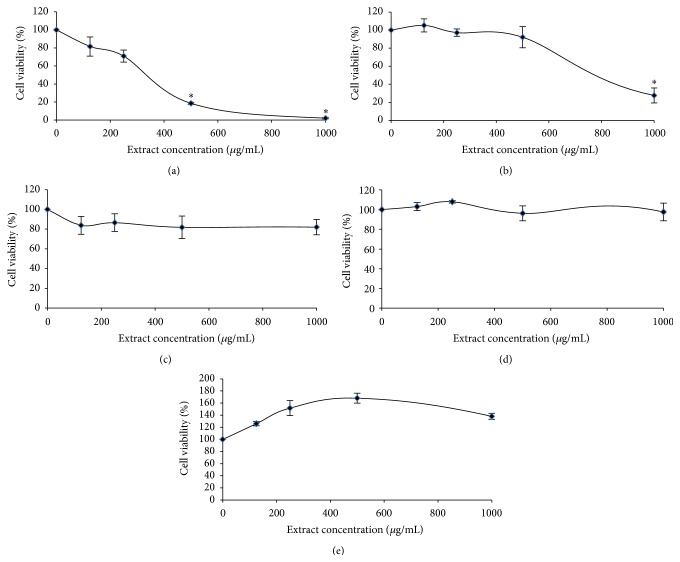
CME exerted selective cytotoxic effect on colorectal carcinoma cells. Percentage viability of (a) HCT 116 colorectal carcinoma cells, (b) HepG2 hepatocellular carcinoma cells, (c) Jurkat E6.1 lymphoblastic leukemic cells, (d) V79-4 Chinese hamster lung fibroblasts, and (e) CCD-841-CoN normal colon epithelial cells following 24-hour treatment with CME. Results are expressed as the means ± SEM of three independent experiments. ^*∗*^ *p* < 0.05. Data were compared between the untreated negative control and the treatment groups by using one-way ANOVA.

CME exerted antiproliferative effects on HepG2 (IC_50_ = 770 *µ*g/mL) and HCT 116 cells (IC_50_ = 340 *µ*g/mL) but not on Jurkat E6.1, V79-4, and CCD-841-CoN cells. The CME antiproliferative effect on HCT 116 and HepG2 cells was found to be concentration dependent. Our results demonstrated CME selective cytotoxicity toward colorectal cancer cells but not toward normal colon epithelial cells. Therefore, HCT 116 cells were used to elucidate the CME mechanism of action on the basis of the potency and selectivity of CME on colorectal cancer cells (Figure 1).

### 3.2. CME Decreased Proliferation of HCT 116 Cells following 24-Hour Treatment

**Figure 2:**
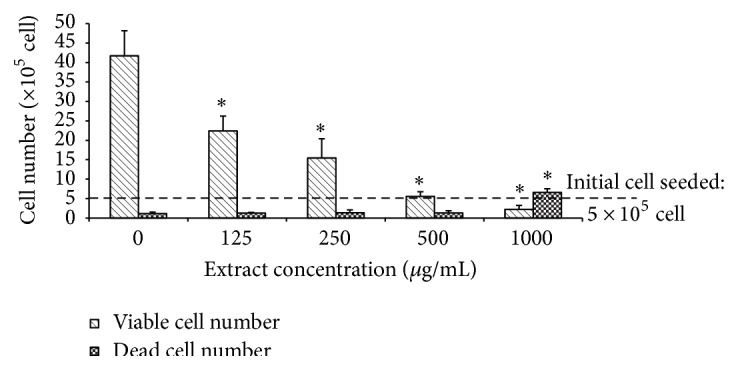
CME inhibited proliferation of HCT 116 cells. Cell count of HCT 116 cells exposed to different concentrations of CME for 24 hours. The results represent the means ± SEM of three independent experiments. ^*∗*^ *p* < 0.05. Data were compared between the untreated negative control and the treatment groups by one-way ANOVA.

CME decreased the proliferation of HCT 116 cells after 24-hour treatment when examined using the trypan blue dye exclusion test. CME treatment as low as 125 *µ*g/mL significantly decreased the HCT 116 viable cells compared with the control population after 24 hours (*p* < 0.05). However, no significant increase in the number of dead cells relative to the control population was observed up to a concentration of 500 *µ*g/mL. This finding suggests that CME exerted its antiproliferative effect primarily by inhibiting the rapid growth of cells with no massive cell death (Figure 2).

### 3.3. CME Induced Cell-Cycle Arrest in HCT 116 Cells

**Figure 3:**
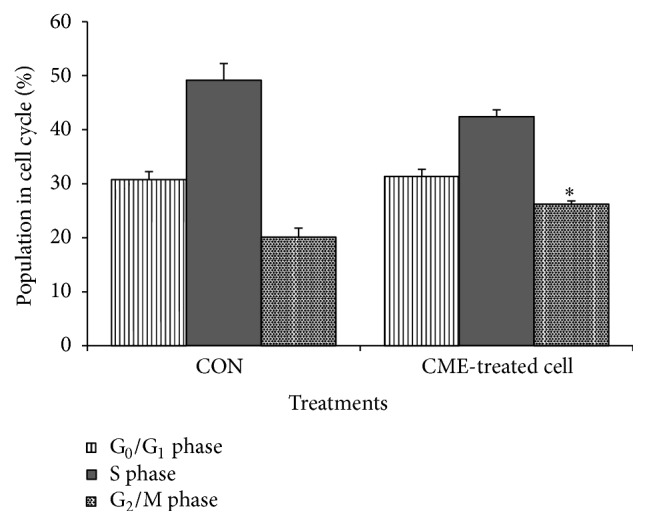
CME induced accumulation of cell population in G_2_/M phase. Cell-cycle analysis of HCT 116 cells treated with 500 *µ*g/mL of CME. The results represent the means ± SEM of three independent experiments. ^*∗*^ *p* < 0.05. Data were compared between the untreated negative control and the treatment groups by one-way ANOVA.

Cell-cycle analysis was performed to elucidate the involvement of cell-cycle arrest in the CME mechanism of action. Following 24-hour treatment, the G_2_/M population of the HCT 116 cells (26.22%  ± 0.6%) was higher than that of the negative control (20.10%  ± 1.7%). This finding suggests that CME affected the cell-cycle distribution, thereby decreasing the proliferation of HCT 116 cells (Figure 3).

### 3.4. CME Induced Apoptosis in HCT 116 Cells

**Figure 4:**
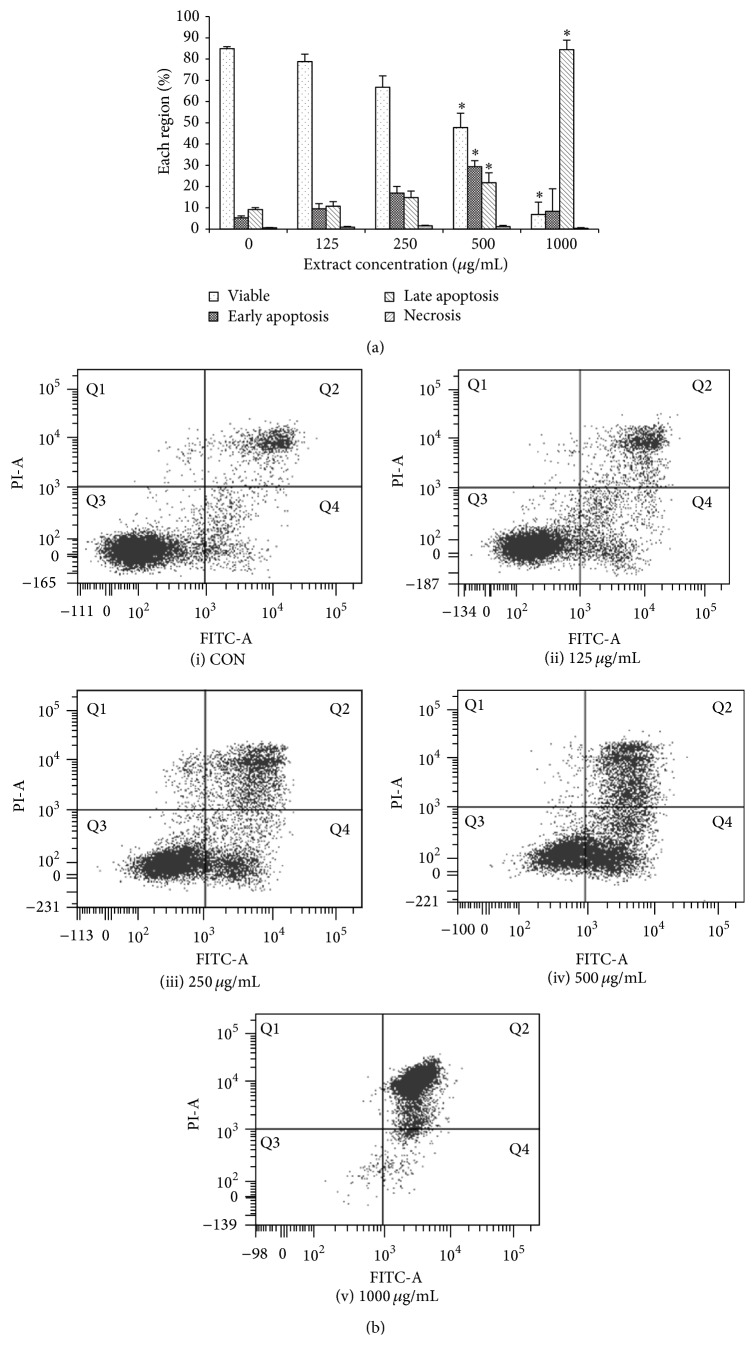
CME induced apoptosis in HCT 116 cells. (a) Mode of cell death investigation on HCT 116 cells treated with different concentrations of CME for 72 hours. (b) Representative dot plots for the (i) control group and the treatment groups treated with (ii) 125, (iii) 250, (iv) 500, and (v) 1000 *µ*g/mL of CME. Data represent the means ± SEM of three independent experiments. ^*∗*^ *p* < 0.05. Data were compared between the untreated negative control and the treatment groups.

Treatment of CME was extended to 72 hours to investigate the mode of cell death in HCT 116 cells. Thus, Annexin V-FITC/PI dual staining was employed. The results of Annexin V-FITC/PI dual staining demonstrated that CME induced apoptosis in a concentration-dependent manner. At a concentration of 500 *µ*g/mL, CME induced 29.30%  ± 4.7% early apoptotic events and 21.77%  ± 2.9% late apoptotic events in the treatment groups, compared with 5.3%  ± 0.8% early apoptotic events and 9.2%  ± 0.9% late apoptotic events in the negative control (*p* < 0.05). Together with the previous data, the current results indicated that CME decreased the proliferation of HCT 116 cells followed by inducing apoptosis (Figure 4).

### 3.5. CME Induced Oxidative Stress in HCT 116 Cells

**Figure 5:**
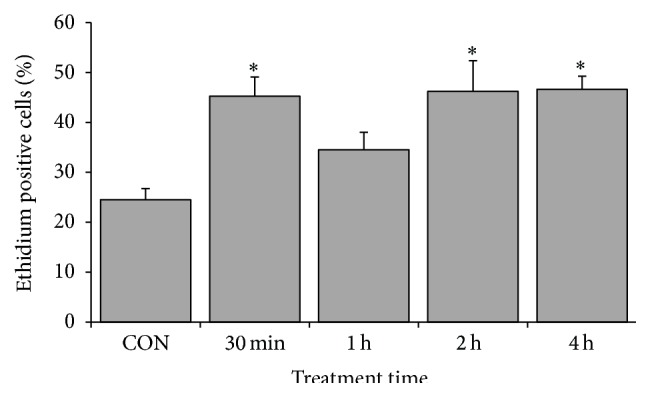
CME induced superoxide anion increment in HCT 116 cells during early time point. Percentages of ethidium positive cells indicate the population with superoxide anion accumulation. The results represent the means ± SEM of three independent experiments. ^*∗*^ *p* < 0.05. Data were compared between the untreated negative control and the treatment groups.

**Figure 6:**
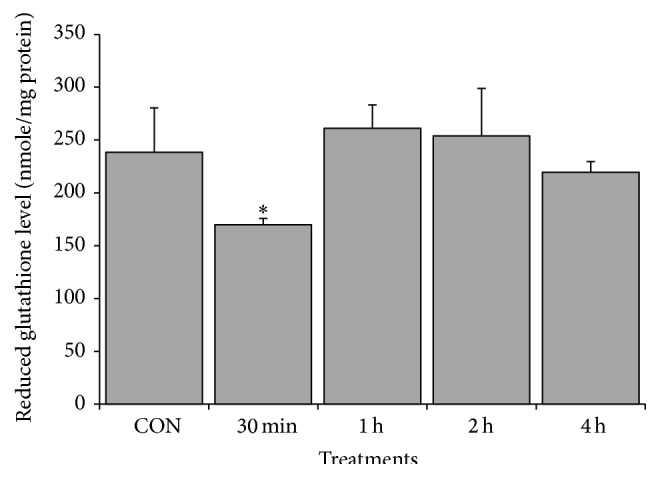
CME induced decrease of intracellular glutathione in HCT 116 cells. HCT 116 cells were treated with 500 *µ*g/mL CME for various time points. The results represent the means ± SEM of three independent experiments. ^*∗*^ *p* < 0.05. Data were compared between the untreated negative control and the treatment groups.

Many studies have demonstrated that oxidative stress is an early signal of apoptosis in cancer cells. Hence, oxidative stress was investigated to understand the mechanism of CME induced apoptosis. Investigation was conducted using 500 *µ*g/mL CME because this concentration induced massive apoptotic events in HCT 116 cells. ROS contents, specifically superoxide anion and intracellular glutathione, were measured to assess the redox status of the CME-treated cells. Our data revealed that 500 *µ*g/mL CME caused an increase in superoxide anion level 1.8-fold as early as 30 minutes and persisted up to 4 hours. Free thiol assessment demonstrated that a 30-minute treatment caused a decrease in glutathione level. The glutathione level in the negative control cells was 283.6 ± 41.0 nmole GSH/mg protein, which significantly decreased to 169.7 ± 6.0 nmole GSH/mg protein after a 30-minute treatment (Figures 5 and 6).

### 3.6. CME Induced DNA Damage in HCT 116 Cells

**Figure 7:**
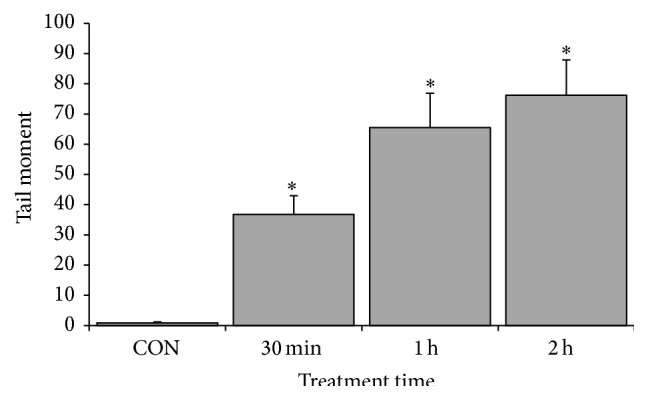
CME induced early DNA damage in HCT 116 cells. HCT 116 cells treated with 500 *µ*g/mL CME at various time points and their tail moments. The results represent the means ± SEM of three independent experiments. ^*∗*^ *p* < 0.05. Data were compared between the untreated negative control and the treatment groups.

**Figure 8:**
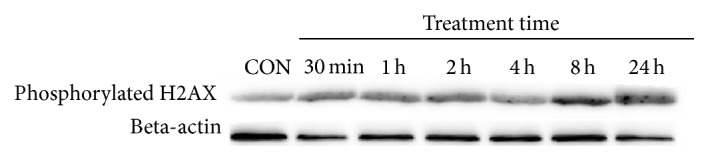
CME increased phosphorylated H2AX level in HCT 116 cells. Level of phosphorylated H2AX in HCT 116 cells treated with 500 *µ*g/mL of CME for various time points, assessed by immunoblot analysis.

Early DNA damage plays a role in halting cell cycle for DNA repair or to trigger apoptosis if the repair mechanism fails. Therefore, alkaline comet assay was conducted, and the phosphorylated H2AX level was assessed to investigate possible involvement of DNA damage in CME-treated cells. Our data showed that the tail moment of the CME-treated cells increased as early as 30 minutes in a time-dependent manner (*p* < 0.05). Immunoblot analysis demonstrated persistent increase in phosphorylated H2AX level up to 24 hours in CME-treated cells compared with the negative control. The overall data emphasized that early DNA damage occurred in CME induced apoptosis (Figures 7 and 8).

### 3.7. CME Induced MMP Loss in HCT 116 Cells

**Figure 9:**
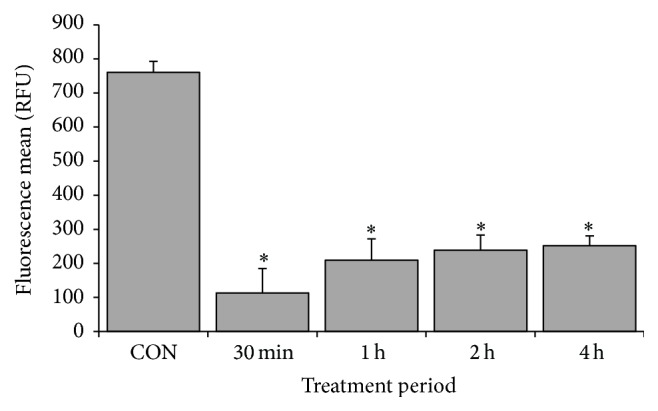
CME induced MMP loss in HCT 116 cells. HCT 116 cells treated with 500 *µ*g/mL CME at various time points and subjected to TMRE staining. The results represent the means ± SEM of three independent experiments. ^*∗*^ *p* < 0.05. Data were compared between the untreated negative control and the treatment groups.

The role of mitochondria in CME induced apoptosis was further investigated in HCT 116 cells. A significant loss in MMP was observed in the HCT 116 cells treated for 30 minutes. The relative fluorescence mean of HCT 116 cells following a 30-minute treatment was approximately sevenfold lower than that of the negative control and remained low for 4 hours. This result highlighted the involvement of mitochondria in CME induced apoptosis (Figure 9).

### 3.8. CME Contained Phenolic Compounds and Saponin

Phytochemical analysis revealed the presence of phenolic compounds and saponin in CME (Table 1).

## 4. Discussion

Pharmacological applications of herbal formulations as alternative medicines do not reflect an emerging practice. Ancestors used herbal formulations for decades without proper scientific evidence. Herbal formulations have gained scientific interest for their pharmacological activities, including anticancer effects [35–40].

This study is the first to report the antiproliferative effect of CME in human colorectal carcinoma cells. Our data showed that CME significantly suppressed the proliferation of colorectal carcinoma cells with minimal effects on normal colon epithelial cells (Figure 1). This finding is important given that the selective cytotoxic activity of the extract toward cancer cells increases its therapeutic value [41, 42]. An expanding list of studies suggested the selectivity of natural products toward cancer cell lines [22, 24, 41–44]. However, to elucidate the possible toxic effects exerted by CME, an* in vivo* study should be conducted.

In the present study, CME exerted an antiproliferative effect, as demonstrated by MTT assay (Figure 1). Cell count showed that the viable cell population decreased in a concentration-dependent manner without massive cell death (Figure 2). The discrepancy observed between the MTT assay and the trypan blue dye exclusion test occurred as a result of different endpoints used in the two different assays [30]. These findings demonstrated that CME exerted an antiproliferative effect during early treatment (24 hours) and induced apoptosis upon longer treatment period (72 hours). A study on the cinnamon extract reported a similar trend of cytotoxicity [22].

Cell-cycle analysis was performed to investigate the possible events that occurred before cell death. CME treatment increased the G_2_/M population in HCT 116 cells. This result suggests the possibility of a cell-cycle arrest during the 24-hour treatment, which explained the reduced proliferation indicated by MTT assay. Studies have proven that active compounds such as 2-hydroxycurcuminoid and subamolide, which were isolated from* Curcuma* spp. and* Cinnamomum* spp., respectively, were capable of arresting cell cycle at G_2_/M phase [22, 45].

Cell death occurs via different mechanisms, such as apoptosis, necrosis, and autophagy. Apoptosis is often involved as a mode of cell death in response to conventional chemotherapeutic agents as well as natural products [18, 19, 29, 44, 46, 47]. Apoptosis is defined as a mechanically driven form of cell death launched in response to various forms of cellular stress [48]. In the current study, we employed Annexin V-FITC/PI dual staining to differentiate both apoptosis and necrosis. CME exhibited apoptosis-inducing properties (Figure 4) at a concentration of 500 *µ*g/mL, which indicated its therapeutic potential in colorectal cancer. Among the genera available in C168 herbal formulation,* Cinnamomum* spp.,* Zingiber* spp., and* Astragalus* spp. are documented to exert antiproliferative effects on human colorectal cancer cells [20–25].* Zingiber officinale* extract was proven to induce cell-cycle arrest and apoptosis in HCT 116 cells [23]. Zerumbone, an active compound extracted from* Zingiber zerumbet*, decreased the proliferation of LS174T, LS180, COLO205, and COLO 320DM cells [24]. Cinnamon, the active compound in* Cinnamomum* spp., decreased the proliferation of DLD-1 human colon cancer cells [20]. Astragalus saponin extracted from* Astragalus membranaceus* suppressed p21 expression, inhibited cyclin-dependent kinase activity, and activated caspase 3 in HT 29 cells, thereby reducing cellular proliferation and promoting apoptosis [25].

In the present study, we observed an early increase in superoxide anion in 30-minute treatment group and a concomitant decrease in the intracellular glutathione level (Figures 5 and 6). Notably, a compensatory increase in glutathione was observed from 1-hour to 4-hour treatment groups (Figure 6). This effect can be attributed to the result of oxidative stress-mediated Nrf-2, ref-1, or NF*κ*B activation that conferred cytoprotectivity to cells [31, 49, 50]. Although there was a compensatory increase in glutathione, the persistence increase in ROS content was speculated to drive the cell to imbalance redox state and generate oxidative stress. Evidence showed that extract from* Zingiber* spp. was capable of inducing ROS-mediated cell death [51–53]. 8-Shogaol and 6-shogaol isolated from* Zingiber* spp. are capable of increasing intracellular ROS, leading to caspase-dependent cell death [51–53].

The double-strand break is one of the most lethal DNA lesions triggering cell-cycle arrest and apoptosis [54, 55]. In the current study, DNA damage was determined using alkaline comet assay and confirmed by detection of phosphorylated H2AX, a biomarker for the DNA double-strand break. We observed DNA damage after CME treatment, as indicated by an increase in tail moment and accumulation of phosphorylated H2AX. Notably, no massive cell death occurred up to the 24-hour time point. Therefore, the DNA damage observed was not a secondary event of DNA fragmentation by caspases during late apoptosis. We also showed that early DNA damage preceded cell-cycle arrest and apoptosis, which occur as much later events. This occurrence suggested that DNA damage can be one of the earliest signals of cell death. Previous studies showed that compounds isolated from* Cinnamomum* spp. can induce DNA damage, as indicated by the upregulation of phosphorylated H2AX [54, 56, 57].

The mitochondrion is an important organelle in an apoptotic event. In the current study, a drastic loss of MMP occurred shortly (30 minutes) after CME treatment (Figure 9), suggesting the involvement of mitochondria in CME induced apoptosis. The loss of MMP was associated with the change in inner mitochondrial membrane permeability. Proteins released from the mitochondria, such as cytochrome c (cyt c) and Smac/DIABLO, triggered caspase-dependent apoptosis, whereas the apoptosis-inducing factor and endonuclease G (Endo G) activated caspase-independent apoptosis [58]. Upstream of apoptotic event, various caspase cascades and cellular machineries are involved in different pathways [59, 60]. To further understand the upstream target of CME, the caspase signaling pathway must be examined.

In the present study, we observed different levels of cytotoxicity on the five cell lines tested. The selectivity of CME can be attributed to the status of p53 in different cells. p53, also known as the guardian of genome, controls a large number of genes mediating cell-cycle arrest, DNA damage recognition, DNA repair, apoptosis, and senescence [61]. When initiated during cellular stress, p53 initiates transcription of p21, leading to cell-cycle arrest. p53 is also known to induce cell death by forming an inhibitory complex with Bcl-XL and Bcl-2, leading to permeabilization of mitochondrial membrane and leakage of cytochrome c [62]. Furthermore, p53 can directly activate Bax and Bak to initiate apoptosis via the mitochondrial mediated pathway [62, 63]. Collectively, p53 plays an important role in cellular growth and cell death in response to stress [61]. HCT 116 and HepG2 cells bear a wild-type p53, whereas Jurkat E6.1 and V79-4 cells contain mutated p53 [64–67]. No data on the status of p53 in CCD-841-CoN cells are currently available. Considering the central role of p53 in stress response, the intact function of p53 may be important in CME induced cell-cycle arrest and apoptosis. However, this hypothesis requires further investigation.

## 5. Conclusion

Our study emphasized the selectivity of CME toward colorectal cancer cells, with the involvement of oxidative stress and DNA damage in CME induced apoptosis. This preliminary study needs further attention to identify the possible mechanisms of action of CME to strengthen its therapeutic value in cancer.

## Figures and Tables

**Table 1:** Phytochemical screening of CME.

Phytochemical content	C168 methanol extract
Alkaloid	−
Triterpenoid	−
Saponin	+
Phenolic compound	+
